# Transmembrane protein 108 involves in adult neurogenesis in the hippocampal dentate gyrus

**DOI:** 10.1186/s13578-019-0272-4

**Published:** 2019-01-11

**Authors:** Zheng Yu, Dong Lin, Yanzi Zhong, Bin Luo, Shengsheng Liu, Erkang Fei, Xinsheng Lai, Suqi Zou, Shunqi Wang

**Affiliations:** 1 Laboratory of Synaptic Development and Plasticity, Institute of Life Science and School of Life Sciences, Nanchang University, Nanchang, Jiangxi China; 2 School of Basic Medical Science, Nanchang University, Nanchang, Jiangxi China; 3 Queen Mary School, Nanchang University, Nanchang, Jiangxi China

**Keywords:** Transmembrane protein 108, Adult neurogenesis, Dentate gyrus, Major depression disorder, Bipolar disorder

## Abstract

**Background:**

Transmembrane protein 108 (Tmem108) is a risk gene of psychiatric diseases including schizophrenia, bipolar disorder and major depression disorder. However, the pathophysiological mechanisms of Tmem108 are largely unknown.

**Results:**

Here we investigated the pathophysiological function of Tmem108 in the hippocampal dentate gyrus by using *Tmem108* mutant mice. Tmem108 highly expressed in the dentate gyrus and CA3 of the hippocampus. Dentate gyrus is a brain region where adult neurogenesis occurs, and aberrant adult neurogenesis in dentate gyrus has been implicated in major depression disorder. Indeed, *Tmem108* mutant mice had lower immobility than wild type mice in tail suspension test and forced swimming test. BrdU and anti-Ki67 antibody staining indicated that adult neurogenesis of the hippocampal dentate gyrus region decreased in Tmem108 mutant mice. qPCR results showed that expression of Axin2, DISC1 and β-Catenin, three dentate gyrus adult neurogenesis related genes in Wnt/β-Catenin signaling pathway, decreased in *Tmem108* mutant mice. Furthermore, Tmem108 enhanced free β-Catenin level in dual luciferase assay.

**Conclusions:**

Thus, our data suggest that Tmem108 increases adult neurogenesis and plays a complexity role in psychiatric disorders.

## Introduction

Adult neurogenesis, the generation of functional neurons from adult neural progenitors, mainly occurs in subventricular zone (SVZ) of the lateral ventricle and subgranular zone (SGZ) of the hippocampal dentate gyrus (DG) [1–3]. Many molecular pathways involve in adult neurogenesis [4], such as Shh, Notch and Wnt signaling pathway. Wnt/β-Catenin signaling pathway has an important role in promoting proliferation of adult neuronal stem cells (or precursor cells) and enhancing differentiation of neuronal precursor cells [4].

Aberrant adult neurogenesis in DG has been implicated in major depression disorder (MDD); in addition, enhancement of adult neurogenesis is regarded as one of the efficient indexes to MDD treatment [5, 6]. MDD, according to world health organization statistics in 2017, as a common mental disorder, affected more than 300 million people worldwide [7]. Severe MDD leads to suicide, and it gives society and family heavy burden and great harm [7].

Transmembrane protein 108 (Tmem108), also named Retrolinkin [8–10], locates on the human chromosome 3q21. Tmem108 expression is found as early as E8.5 in central nervous system [11, 12] and it plays an important role in central nervous system [8–10, 13]. Genome-wide association study (GWAS) found that TMEM108 is a susceptibility gene of psychiatric disorder, including schizophrenia, bipolar disorder (BPD) and MDD [14–17].

In the process of characterizing function of Tmem108 in psychiatric disorders, we found that Tmem108 is developmentally regulated and is required for glutamatergic transmission in DG [13]. We speculated that Tmem108 involved in MDD by regulating adult neurogenesis. In this study, the results showed that Tmem108 played a complexity role in psychiatric disorders, *Tmem108* mutant mice had lower immobility than wild type mice in depression-like behavior tests, and adult neurogenesis of the hippocampal DG in *Tmem108* mutant mice decreased. Further study indicated that Tmem108 could regulate adult neurogenesis by affecting Wnt/β-Catenin signaling pathway.

## Materials and methods

### Materials

*Tmem108* mutant (*Tmem108*-*LacZ*) mice were gifts from Dr. L. Mei, which were described previously [12, 13]. Mice were housed in a room 12-h light/dark cycle with ad libitum access to water and rodent chow diet.

The animal protocols in this study were approved by Nanchang University Medical Sciences Committee for research in vertebrate animals, in accordance with EN Directive 2010/63/EU on the protection of animals used for scientific purposes [18]. For in vivo experiment, surgery was performed under sodium pentobarbital anesthesia (50 mg/kg, ip injection), and all efforts were made to minimize suffering. After terminal experiments, mice were euthanized by carbon dioxide inhalation followed by cervical dislocation.

### Reagents

X-gal (5-Bromo-4-chloro-3-indoly β-d-galactopyranoside) was purchased from Sigma-Aldrich (cat. #: B4252, 30 mg/ml for staining). BrdU (5-Bromo-2′-deoxyuridine) was purchase from Sigma-Aldrich (cat. #: B5002, 5 mg/ml for ip injection).

Antibodies information was as follows: anti-BrdU (Rat, Accurate Chemical & Scientific Corporation, cat.#: OBT0030; 1:1000 for staining); anti-Ki67 (Mouse, BD Biosciences cat.#: 550609; 1:1000 for staining); Alexa Fluor 488 Donkey anti-rat lgG (Thermo Fisher Scientific, cat.#: A-21208; 1:1000 for staining), Alexa Fluor 568 goat anti-rabbit lgG (Thermo Fisher Scientific, cat.#: A11011; 1:1000 for staining); anti-β-Actin antibody (Rabbit, Santa Cruz Biotechnology cat.#: sc-1616-R; 1:2000 for blotting); anti-Tmem108 antibody (Rabbit, 1:1000 for blotting) was kindly provided by Dr. J. Liu [8]. Polyclonal horseradish peroxidase (HRP)-conjugated goat anti-rabbit IgG (cat. #: 32260) and goat anti-mouse IgG (cat. #: 32230) secondary antibodies were purchased from Pierce Thermo Fisher Scientific (1:2000 for blotting).

The construct pFlag-cmv1-Wnt3a was described, previously [18]. The construct pFlag-cmv1-Tmem108 was described previously [13]. The vector p3×Flag-cmv-24 was kept by our lab.

### Cell line and culture condition

HEK293 cell line was kindly provided by Dr. S. Luo and the cells were maintained in Dulbecco’s modified Eagle medium (DMEM; 4.5 g/l d-glucose, Thermo Fisher Scientific) supplemented with 10% fetal bovine serum (Thermo Fisher Scientific) and 1 × Penicillin–Streptomycin Solution (Thermo Fisher Scientific) in tissue culture dishes in a humidified incubator at 37 °C with an atmosphere of 95% air and 5% carbon dioxide.

### In vitro transfection and dual luciferase reporter assay

All transfection was performed using lipofectamine 3000 transfection reagent, according to the manufacturer’s instructions (Thermo Fisher Scientific). Firefly luciferase reporter vectors (TOP-Flash and Fop-Flash) were generous gifts form Dr. L. Mei, as well as Renilla luciferase vector pRL-TK, which utilized as a transfection internal control.

HEK293 cells were seeded into 24-well plate. Total 760 ng plasmids DNA/well were transfected, using ratio of p3×Flag-cmv-24 or pFlag-cmv1-Wnt3a or pFlag-cmv1-Tmem108: Firefly luciferase reporter vector (TOP-Flash or Fop-Flash): pRL-TK = 50: 25: 1. Each reaction was in triplicate wells.

### RNA extraction and qPCR

Total RNA from mice tissues (wild mice n = 3) and/or HEK293 cells was extracted using TRIzol Reagent (Invitrogen) according to the manufacturer’s instructions. qPCR was performed as described previously [19]. The relative expression values were calculated relative to Gapdh or β-Actin by using the 2^−ΔCT^ methods. Results were normalized to the control using the Microsoft Excel program. The qPCR primer sets were listed in Table 1.

**Table 1:** Primer sequence used in qPCR assays

Gene name	Forward primer sequence (5′–> 3′)	Reverse primer sequence (5′–> 3′)
Tmem108	CCTGAGCTACTGGAACAATGCC	CAGTGTCTCGATAGTCGCCATTG
Gapdh	CATCACTGCCACCCAGAAGACTG	ATGCCAGTGAGCTTCCCGTTCAG
β-Actin	CATTGCTGACAGGATGCAGAAGG	TGCTGGAAGGTGGACAGTGAGG
Disc1	TGGTCGAGGATGGCGATTACGA	AGAGCAGGTTGCTGTGAAGGCA
β-Catenin	GTTCGCCTTCATTATGGACTGCC	ATAGCACCCTGTTCCCGCAAAG
Apc	GTGGACTGTGAGATGTATGGGC	CACAAGTGCTCTCATGCAGCCT
Gsk3β	GAGCCACTGATTACACGTCCAG	CCAACTGATCCACACCACTGTC
PI3K	CAAACCACCCAAGCCCACTACT	CCATCAGCAGTGTCTCGGAGTT
Axin1	GTCCAGTGATGCTGACACGCTA	GCCCATTGACTTGGATACTCTCC
Axin2	ATGGAGTCCCTCCTTACCGCAT	GTTCCACAGGCGTCATCTCCTT

### BrdU injection and BrdU staining

BrdU injection (wild type mice n = 5, *Tmem108* −/− mice n = 5) and staining was performed as described previously [20] with minor modifications. In briefly, first, BrdU was dissolved in 0.9% NaCl and ip injected three times at 1 h intervals at 100 mg/kg mice weight. Next, 24 h following the last injection, the brain was perfused and fixed with 4% paraformaldehyde (PFA). In order to denature DNA, frozen sections of the mice brain tissue were incubated in 1 M HCL for 30 min at room temperature, and acid was neutralized by rinsing section three times with PBST (0.1 M PBS (phosphate buffer saline) containing 0.1% Triton X-100). After blocked in blocking buffer (containing 5% goat serum, 2% BSA, 0.1% Triton X-100, 0.1% NaN_3_ in PBS, pH 7.4) for 1 h at room temperature, the section was incubated with BrdU primary antibody in antibody buffer (containing 2% goat serum, 1% BSA, 0.1% NaN_3_ in PBS, pH 7.4) for 72 h at 4 °C. And after rinsed three times with PBST, the sections were incubated with fluorochrome-conjugated secondary antibody in antibody buffer in the dark for 2 h at room temperature. Next, the sections were incubated with DAPI for 5 min and rinsed with PBS. At last, the sections were transferred to slides by a soft brush and were mounted with coverslips. The images were captured by inverted fluorescence microscope (Olympus FSX100).

### Immunohistochemistry (IHC)

The mice brain (wild type mice n = 4, *Tmem108* −/− mice n = 4) was perfused and fixed with 4% PFA, and the frozen sections were used for IHC similar to BrdU staining protocols as mentioned above with minor modification. Briefly, after washed three times with PBS, the fresh sections were blocked for 1 h at room temperature. Then, the sections were incubated with primary antibody overnight at 4 °C. Next, the sections were rinsed three times with PBS, and incubated with secondary antibody in the dark for 2 h at room temperature. Finally, the sections were transferred to slides, and mounted with coverslips. The images were captured by inverted fluorescence microscope (Olympus FSX100).

### X-gal staining

The mice (*Tmem108* +/− mice n = 3) brain was perfused and fixed with 4% PFA, and the fresh frozen sections were used for X-gal staining. Firstly, the sections were washed with PBS containing 2 mM MgCl_2_ for 10 min at 4 °C, and permeabilized with detergent rinse (2 mM MgCl_2_, 0.01% sodium deoxycholate, 0.02% NP-40 in 0.1 M phosphate buffer, pH 7.4) for 10 min at 4 °C. Next, the sections were stained with staining solution (5 mM potassium ferrocyanide, 5 mM potassium ferricyanide, 2 mg/ml X-gal in detergent rinse) overnight at 37 °C. Then, after washed with PBS, the sections were added mount medium, and mounted with coverslips. Finally, the images were Z-stack taken by inverted fluorescence microscope (Olympus FSX100).

### Western blot

Lysates of tissues were prepared in modified RIPA buffer (1 × DPBS, 0.1% SDS, 0.5% sodium deoxycholate, 1% NP-40, 1 mM PMSF, 1 mM EDTA, 1 mg/ml aprotinin, leupeptin, and pepstatin A protease inhibitors). Samples were resolved by SDS-PAGE and transferred onto nitrocellulose membranes, which then were blocked with blocking buffer containing 5% nonfat dry milk in TBST buffer (20 mM Tris, 150 mM NaCl and 0.1% Tween 20) for 1 h at room temperature. Then, the membranes were washed with TBST buffer for 10 min three times before it was incubated with primary antibody overnight at 4 °C. After washing three times with TBST, the membranes were incubated with HRP-coupled secondary antibody in blocking buffer for 1 h at room temperature. The immunoreacted bands were captured by enhanced chemiluminescence system (Bio RAD).

### Behaviors test

In behavioral analysis, 10- to 12-week-old male mice were carried out by investigators unaware of mice genotype, and the mice were handled for habituation for 2 days before behavioral tests.

Open field test (wild type mice n = 12, *Tmem108* −/− mice n = 10) was performed as described previously [13, 21]. In briefly, mice movements in the chamber (50 × 50 × 80 cm) were monitored by an overhead camera and tracking software (Video tracking interface version 1.85, Med Associate Inc.) for 30 min. The mice used in open field test were reused one more time in forced swimming test (TST) or tail suspension test (FST).

For TST, mice (wild type mice n = 12, *Tmem108* −/− mice n = 15) were hung approximately 15 cm away from the bottom of the testing box for 6 min. The immobility in the last 4 min was measured by sensory element controlled by computer software (Activity monitor version 6.02, Med Associate Inc.).

For FST, mice (wild type mice n = 17, *Tmem108* −/− mice n = 10) were forced to swim in a 2-l beaker filled with about 15 cm height water for 6 min. Mice movement were monitored by horizontal camera and tracking software (Video freeze version 2.5.5.0, Med Associate Inc.). The immobility in the last 4 min was used for statistics.

### Statistical analysis

Statistical analysis was conducted by GraphPad Prism 6.01. All data in each group were presented as mean with SEM (standard error of the mean) and data between groups were analyzed by two-tailed student t-test. Difference was considered if the p value < 0.05.

## Results

### High Tmem108 expression area in the brain

**Fig. 1:**
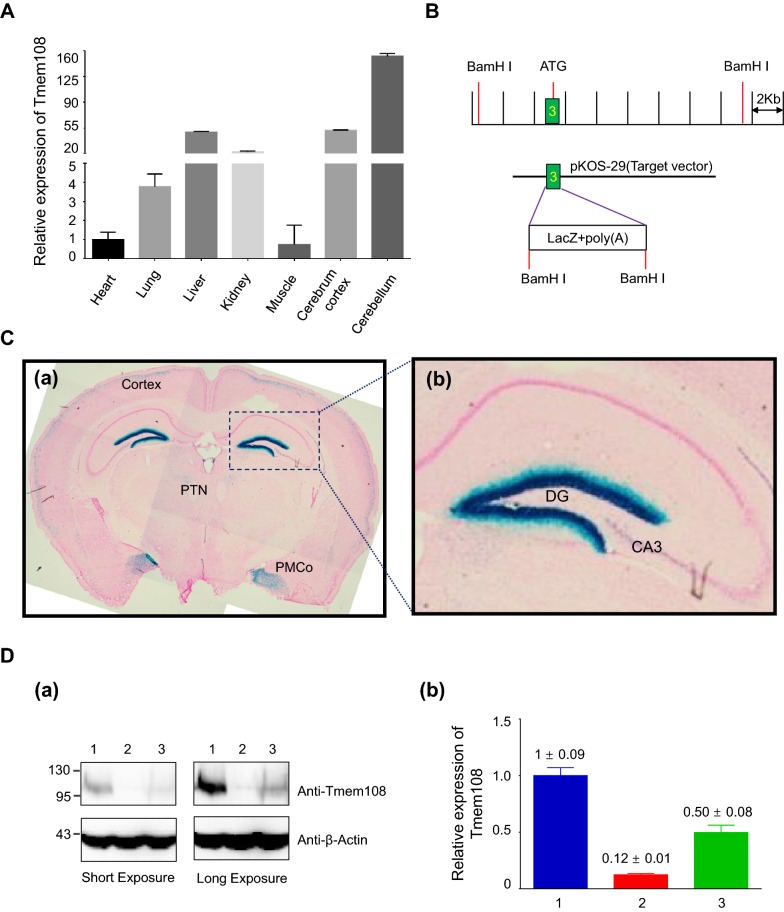
High Tmem108 expression area in mice brain. **A** Tmem108 relative expression in different tissues in adult wild type mice (Gapdh expression as internal control, Tmem108 expression in heart was defined as one, wild type mice n = 3). **B** *Tmem108* mutant mice targeting strategy; the initiation code of *Tmem108* localizes in the exon 3, and *LacZ* gene is inserted into the downstream of *Tmem108* promotor. **C** LacZ staining of *Tmem108* mutant mouse (Tmem108 +/−) hippocampus; PMCo: posteromedial cortical amygdaloid nucleus, PTN: Parafascicular thalamic nucleus. **D** *Tmem108* levels in hippocampus were evaluated by Western blotting assay; **D** (a) Representative image of Western blotting assays, **D** (b) Statistics of Tmem108 expression from Western blotting assay; 1: wild type mice n = 3; 2: Tmem108 knockout mice homozygous (Tmem108 −/−) n = 3; 3: Tmem108 knockout mice heterozygous (Tmem108 +/−) n = 3

We first evaluated the relative expression of Tmem108 in different tissues, RT-qPCR was used to check Tmem108 expression in adult wild type mice (Fig. 1A). As a classic internal control, Gapdh expression was used to normalize Tmem108 expression in seven selected tissues (heart, lung, liver, kidney, muscle, cerebrum cortex and cerebellum). Tmem108 expression in heart was defined as one, Tmem108 has a highly expression in liver, kidney, cerebrum cortex and cerebellum, comparing with the expression in heart and muscle (over 20 times).

LacZ staining was also used to examine Tmem108 expression in the brain. *Tmem108* mutant mice targeting strategy was described before [13], in brief words, LacZ cassette including both stop code and a poly adenylation termination signal was inserted into the downstream of Tmem108 promoter (Fig. 1B). We found that Tmem108 had high expression in posteromedial cortical amygdaloid nucleus (PMCo), parafascicular thalamic nucleus (PTN), cortex, DG and CA3 of the hippocampus than other regions in brain coronal section slides (Fig. 1C).

Western blot confirmed Tmem108 expression between *Tmem108* mutant mice and wild type mice (Fig. 1D). Nearly 90% of Tmem108 were successfully knockout in homozygous mutant mice (*Tmem108* −/−), and Tmem108 expression in heterozygous mutant mice (*Tmem108* +/−) was consistent with theoretical estimation (50% knockout).

### Decrease of immobility in *Tmem108* mutant mice

**Fig. 2:**
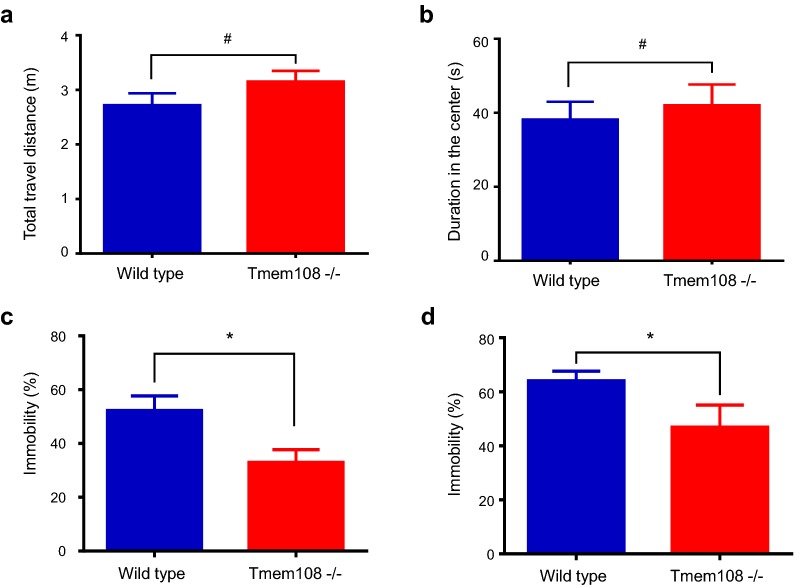
*Tmem108* mutant mice had lower immobility. **a** *Tmem108* mutant mice locomotor activity was not different from wild type mice in open field test (total travel distance was no difference). **b** *Tmem108* mutant mice did not have anxious behavior in open field test (duration in the center was no difference, wild type mice, n = 12, Tmem108 −/− mice, n = 10). **c** *Tmem108* mutant mice had lower immobility in tail suspension test (TST, wild type mice n = 12, Tmem108 −/− mice n = 15). **d** *Tmem108* mutant mice had lower immobility in forced swimming test (FST, wild type mice n = 17, Tmem108 −/− mice n = 10) (^#^p > 0.05, *p < 0.05)

As shown in Fig. 2, three kinds of animal behavior assays were carried out to investigate whether depression-like behaviors were altered in *Tmem108* mutant mice, First, both total travel distance and duration in the center in open field test were not changed in *Tmem108* mutant mice (Fig. 2a, b), which suggest that Tmem108 does not alter mice locomotor activity and anxious behavior. Then, both FST and TST were employed in depression-like tests (Fig. 2c, d), *Tmem108* mutant mice exhibited lower immobility than wild type mice in both assays. These behavior tests suggest that depression-like behaviors in mice reduce in *Tmem108* mutant mice.

### Decrease of DG adult neurogenesis in *Tmem108* mutant mice

**Fig. 3:**
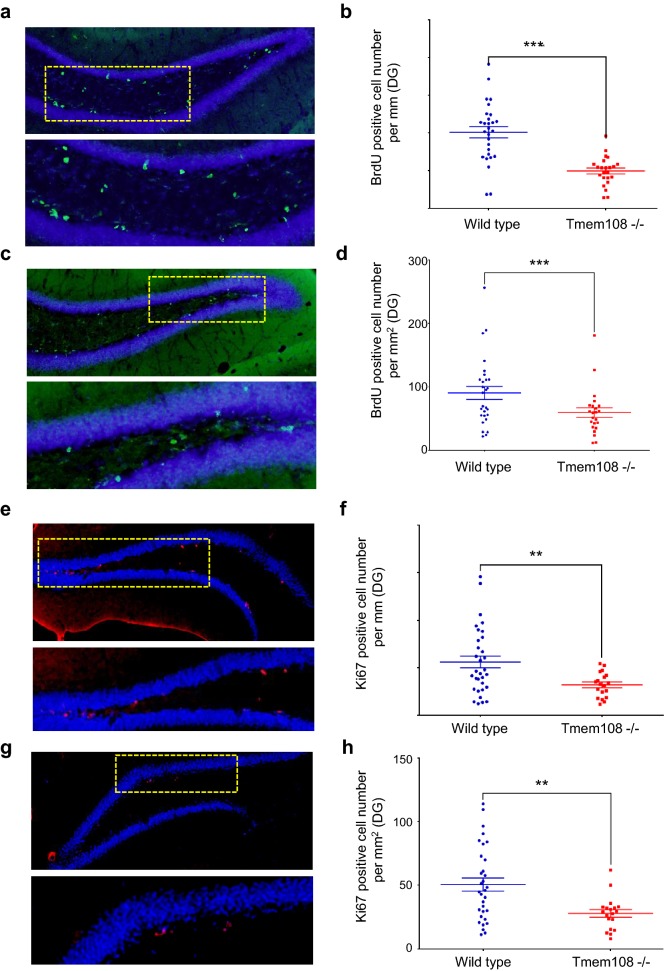
Loss of Tmem108 decreased DG adult neurogenesis. Representative images of BrdU assay, the low panel was from the rectangular area of the upper panel in **a** (wild type mice n = 5) or **c** (Tmem108 −/− mice n = 5); 2-month mice were injected ip with 300 mg/kg BrdU 24 h before sacrifice; incorporated BrdU was detected in sections by IHC using an anti-BrdU antibody (green), nuclei were counterstained with DAPI (blue). Quantification of BrdU positive cell number per mm DG inner edge (**b**) and per square mm DG region (**d**). Representative images of anti-Ki67 staining (red) from 2-month mice, the low panel was from the rectangular area of upper panel in **e** (wild type mice n = 4) or **g** (Tmem108 −/− mice n = 4); nuclei were counterstained with DAPI (blue). Quantification of Ki67 positive cell number per mm DG inner edge (**f**) and per square mm DG region (**h**) (**p < 0.01, ***p < 0.001)

Figure 3 shows DG adult neurogenesis by BrdU staining assay and anti-Ki67 staining separately. Mice were injected ip with BrdU 24 h before sacrifice, and incorporated BrdU was detected in coronal sections by immunohistochemistry using an anti-BrdU antibody (Fig. 3a, c). BrdU positive cell numbers in the inner granule cell layer edge of the DG (per millimeter) and/or in DG granule cell layer (per square millimeter) decreased in *Tmem108* mutant mice (Fig. 3b, d), which suggests that ablation of Tmem108 decreases DG neuronal progenitor cells proliferation.

Anti-Ki67 staining confirmed the result suggestion in BrdU assay (Fig. 3e, g). Without controversy, Ki67 positive cells in granule cell layer of the DG also decreased in *Tmem108* mutant mice (Fig. 3f, h). These indicate that Tmem108 increases adult neurogenesis in mice.

### Tmem108 affects Wnt/β-Catenin signaling pathway

**Fig. 4:**
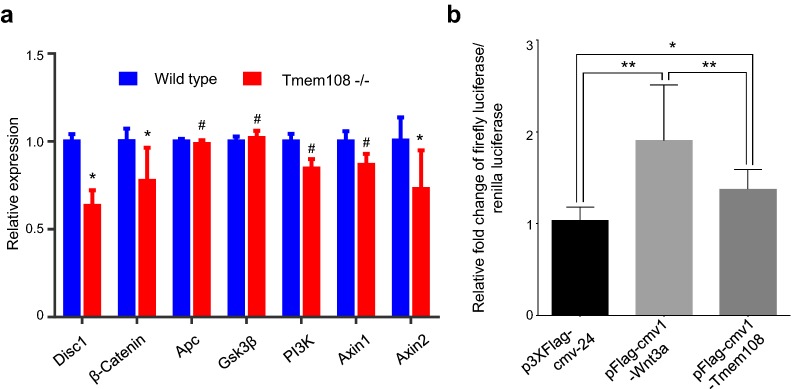
Tmem108 affected Wnt/β-Catenin signaling pathway. **a** Expression profiling of Wnt signaling pathway by qPCR. Wnt signaling pathway was disturbed in hippocampus of *Tmem108* mutant mice; Gapdh mRNA expression was used as an internal control, mRNA expression relative to Gapdh in wild type was defined as one (wild type mice, n = 5, Tmem108 −/− mice, n = 4, ^#^p > 0.05, *p < 0.05). **b** In HEK293 cell culture, Tmem108 increased free β-Catenin level in Dual luciferase assay. Relative TOP-Flash Firefly Luciferase (TFL1)/Renilla Luciferase (RL1) = Raw TFL1/Raw RL1; Relative FOP-Flash Firefly Luciferase (FFL2)/Renilla Luciferase (RL2) = Raw FFL2/Raw RL2; and final relative Firefly Luciferase (FL)/Renilla Luciferase (RL) = (Raw TFL1/Raw RL1)/(Raw FFL2/Raw RL2). Fold changes of FL/RL at p3×Flag-cmv-24-treated condition is normalized as 1, and fold changes of FL/RL at pFlag-cmv1-Wnt3a-treated or pFlag-cmv1-Tmem108-treated condition is normalized to FL/RL at p3×Flag-cmv-24-treated condition

Lastly, expression profiling of Wnt/β-Catenin signaling pathway were evaluated by RT-qPCR. On the premise of Gapdh expression as reference control, Axin2, Disc1 (Disrupted-in-Schizophrenia 1) and β-Catenin decreased in *Tmem108* mutant mice hippocampus (Fig. 4a), which suggests that Wnt/β-Catenin signaling pathway is disturbed in the hippocampus of *Tmem108* mutant mice.

To further determine whether Tmem108 involves classic Wnt/β-Catenin signaling pathway, dual luciferase assay was carried out in vitro. In HEK293 cell culture, Tmem108 increased free β-Catenin level in the assay, though the activation was lower than the positive control Wnt3a (Fig. 4b), which suggests that Tmem108 enhances Wnt/β-Catenin signaling pathway.

These data suggest that Tmem108 does not only increase adult neurogenesis in the hippocampal DG, but also affects Wnt/β-Catenin signaling pathway. And furthermore, *Tmem108* mutant mice behaviors in depression-like tests indicate that Tmem108 plays a complexity role in psychiatric disorders.

## Discussion

In this study, the results indicate that Tmem108 highly expresses in DG and CA3 region of the hippocampus, and it acts as a positive signal in adult neurogenesis. Adult neurogenesis mainly occurs in SVZ and SGZ of DG region [1–3]. Because of convenient detection and easy quantification, DG region was frequently used to evaluate adult neurogenesis [5, 22, 23]. One of the hypotheses on MDD pathogenesis is decrease of adult neurogenesis [5, 23]. Accordingly, MDD usually exhibits deficiency on adult neurogenesis of DG region. Because adult neurogenesis decreases in *Tmem108* mutant mice, *Tmem108* mutant mice may be linked to depression.

*Tmem108* mutant mice exhibit low immobility in both forced swimming test and tail suspension test, which look like anti-depression-like or mania-like behaviors. Therefore, adult neurogenesis decrease and anti-depression behavior are not consistent in *Tmem108* mutant mice. The prevalent perspectives in MDD research hold that immobility is interpreted as a negative mood behavior [5, 24], and others do not agree with the opinion [25, 26]. *Tmem108* mutant mice may habituate quickly, or have weak persistence in stress condition, and consequently, *Tmem108* mutant mice show low immobility in stress environment.

In GWAS analysis, Tmem108 is related with BPD, MDD and schizophrenia [14–16], which share many similar behaviors. Low immobility may be a reflection of schizophrenia or BPD. It has been found that *Tmem108* mutant mice have deficiency in prepulse inhibition test [13], which is a symbol of schizophrenia. Furthermore, in GWAS analysis, the p value in BPD and schizophrenia GWAS analysis are 0.00328 and 0.0024, separately [15, 16], much higher than p value (0.0000148) in MDD [14]. BPD, formerly called mania depression, one of the hallmarks is the alternation of depression period and mania period [27]. Therefore, Tmem108 may play a complexity role in psychiatric disorders.

Low immobility in *Tmem108* mutant mice may be an abnormal physiological manifestation induced by stress condition. To confirm the hypothesis, more behavior tests should be compared in normal condition with in stress condition. Whatever the cause of decrease of *Tmem108* mutant mice immobility, it is unquestionable that adult neurogenesis in *Tmem108* mutant mice decrease.

We wonder whether Tmem108 regulates adult neurogenesis by affecting Wnt//β-Catenin signaling pathway. Wnt/β-Catenin signaling is a canonical pathway in control cell proliferation and differentiation, and it also involves adult neurogenesis [28–30]. In fact, several genes expression of Wnt/β-Catenin signaling pathway decreased in *Tmem108* mutant mice, such as β-Catenin, Axin2 and Disc1. Furthermore, in vitro dual luciferase assay suggests that Tmem108 increases free β-Catenin level. Free β-Catenin, as an intracellular signal transducer, is the main downstream executor of Wnt/β-Catenin signaling pathway [31].

In conclusion, our study demonstrated that Tmem108 involves in adult neurogenesis and it may regulate Wnt/β-Catenin signaling pathway to affect adult neurogenesis. Furthermore, abnormal behaviors in *Tmem108* mutant mice indicated that Tmem108 plays a complexity role in psychiatric disorders.

## Abbreviations

BPD: bipolar disorder
DG: dentate gyrus
FST: forced swimming test
GWAS: genome-wide association study
IHC: immunohistochemistry
MDD: major depression disorder
PFA: paraformaldehyde
SGZ: subgranular zone
SVZ: subventricular zone
Tmem108: transmembrane protein 108
TST: tail suspension test
X-gal: 5-bromo-4-chloro-3-indoly β-d-galactopyranoside
